# Barriers and enablers of adverse drug event reporting in South Africa: a cross-sectional survey of medicine users

**DOI:** 10.1186/s12889-025-24409-1

**Published:** 2025-09-25

**Authors:** Nokukhanya Ncube, Martha S. Lubbe, Hanlie Steyn, Nkengafac V. Motaze

**Affiliations:** Medicine Usage in South Africa, Faculty of Health Sciences, North-West University, Potchefstroom, South Africa

**Keywords:** Adverse drug events; barriers, Enablers, Pharmacovigilance, Consumers

## Abstract

**Background:**

Spontaneous reporting is the most frequently used method to collect data on adverse drug events (ADEs). To promote ADE reporting and enhance medicine safety, it is important to understand enablers and barriers to ADE reporting. This study described medicine users’ practices and previous experiences with reporting ADEs, including barriers and enablers to reporting ADEs in South Africa.

**Methods:**

This was a cross-sectional, analytical study in which adults residing in South Africa were requested to complete an online questionnaire.

**Results:**

The majority of participants (70.5%, *n* = 203; *N* = 288) indicated that they experienced ADEs in the past and 77.0% (*n* = 181; *N* = 235) of these events occurred after the use of medication. Only 59.1% (*n* = 120; *N* = 203) of participants who experienced ADEs, reported them. There was no difference in reporting frequency between healthcare professionals (HCP) and non-HCPs (*p* > 0.9). Furthermore, 78.3% (*n* = 94; *N* = 120) of participants reported ADEs to their doctor.

The main enabler for reporting ADEs indicated by both HCP and non-HCPs was that reporting could help to keep others safe. The most cited barrier to ADE reporting for both HCP and non-HCPs was the assumption that the side effects would go away once the medication was stopped.

**Conclusions:**

A large proportion of participants who experienced ADEs reported them to their doctors, and reporting of ADEs was similar between HCPs and non-HCPs. The barriers and enablers to reporting ADEs were similar for HCPs and non-HCPs. These results could help in planning awareness-raising campaigns aimed at improving ADE reporting in South Africa.

## Background

Adverse drug events (ADEs) are reported to be a frequent reason for unanticipated hospitalisation and fatalities [1]. Adverse drug events can place a significant financial burden on the healthcare system and are reported to be a key driver of unscheduled hospital admissions and emergency room visits [2]. The South African Health Products Regulatory Authority (SAHPRA) defines an ADE as “any untoward medical occurrence that may present during treatment with a medicine, but which does not necessarily have a causal relationship with this treatment. An adverse event can be any unfavourable and unintended sign, symptom or disease temporarily associated with the use of a medicine, whether considered related to the medicine or not” [3]. A study discovered that the median prevalence rate of ADEs during hospitalization in general patient populations was 7.8%, with an average of 45.0% of ADEs being avoidable and 25.0% of ADEs classified as serious [4].

It is estimated that patients in low-income countries face medication-related injury at a rate that is at least two times higher than those in high-income countries [5]. However, the rate of reporting is low. This could be explained by underreporting, which has been identified as one of the primary challenges to pharmacovigilance in Africa [6]. A review of studies on ADEs conducted in Ethiopia, Morocco, Nigeria, South Africa, Tunisia, and Uganda found that, on average, 8.4% of patients stated that they experienced an ADE during hospitalisation. In contrast, about 2.8% of patients were admitted to health facilities due to ADEs [7].

A spontaneous report is a report to a medicine manufacturer, regulatory agency, or other association that can be made by healthcare providers, patients or consumers. This report details a possible ADE which occurred in a patient who has used a specific medicine, and this information is not solicited (e.g., not collected as part of any study or active surveillance) [3]. Spontaneous reporting is a low-cost, adaptable, and highly efficient information-gathering technique through which both consumers and HCPs can report ADEs [8]. The primary benefits of spontaneous reporting in pharmacovigilance are that reports can be related to any medicines, they can be from any level of healthcare services, they can be focused on specific medicines and observed ADEs, facilitate coding of the reported ADEs, reveal the timing and progression of the reactions and can also reveal the existence or absence of additional risk factors and the patient’s medical background [9].

Despite the obvious advantages mentioned above some limitations of spontaneous reporting have been noted to be the incomplete reports submitted by consumers [10]; reporting of ADEs can be influenced by individuals such as family members, friends, healthcare providers as well as information found online and on social media [11].

In South Africa, HCPs and the general public can report ADEs to SAHPRA electronically (via the Med Safety App or email) and telephonically. Patient reporting has been shown to play a significant role in the pharmacovigilance system, as reports of adverse events increased by about 30% in a year with the involvement of both patients and those who look after them [12]. Adverse drug events that patients report have been found to contain clinical data that is comparable to reports from healthcare professionals (HCP) and patient reports have the added advantage of providing thorough accounts of the ADE experience and its effects on their daily lives [13]. When combined with HCP reporting, patient reporting enhances the depth of to the reports that are received, making them valuable for pharmacovigilance [14].

The Medicines and Related Substances Act 101 of 1965 [15], mandates the South African Health Products Regulatory Authority (SAHPRA) to ensure that health products and their use are regulated in South Africa. The vigilance unit of SAHPRA oversees the vigilance activities, which include the gathering and assessment of reported ADEs [3]. The minimum requirements for reporting an ADE are the presence of an involved patient, the requirement that a medicinal product be involved, the suspicion of an ADE, and the reporter’s contact information [16]. Although South Africa has an established pharmaceutical surveillance system and currently contributes ADE data to the World Health Organization’s (WHO) Programme for International Drug Monitoring [17], underreporting of ADEs in South Africa is still a problem [18].

Lack of reporting of ADEs makes it difficult to promptly recognise medicine-related risks to patient safety. Therefore, it is critical to understand the factors that act as barriers and enablers to ADE reporting to promote learning and enhancement of patient safety [19]. In a systematic review of variables affecting ADEs reporting, it was found that ADE reporting was hindered by various factors, including lack of awareness, ambiguity regarding who must report, a challenging reporting process, a lack of feedback, or a bad reporting experience [20]. Public participation results when consumers have a platform to share their experiences and receive acknowledgement of their reports [21].

This study aimed to describe medicine users’ practices and previous experience with reporting ADEs, including the barriers and enablers to reporting ADEs in South Africa. The knowledge gained from this study will provide insights into the barriers that medicine users face in reporting ADEs and guide the actions that enhance enablers of ADE reporting by consumers in South Africa.

## Methods

We conducted a cross-sectional analytic study using a self-administered, online, structured questionnaire as the data collection tool, which was generated using questionnaires from published studies with similar aims [22–26]. The setting for the study was both the private and public healthcare sector in all nine provinces of South Africa and the target population for this study included all adults currently residing in South Africa The survey’s 32 questions were arranged in the following sections: Section A: Socio-demographic information; Section B: Awareness of adverse drug events; Section C: Attitudes regarding adverse drug events and adverse drug events reporting; Section D: Practices and experience with reporting adverse drug events; Section E: Barriers to reporting adverse drug events. The questionnaire’s section A, C, D and E are presented in this paper. The face validity of the questionnaire was assessed by a statistician, and its relevance to the objectives of the study was assessed by specialists from the Medicine Usage in South Africa Scientific Committee. Every participant was given the same questionnaire to complete.

Participants were recruited via a news article published on 18 April 2023 on the News24 online platform in English and translated to Afrikaans and published on Netwerk24 [27]. The article, which was accessible to all individuals with access to these publishing platforms, contained a link to direct the readers to SurveyMonkey^®^ [28] to complete the survey. The survey was launched on 18 April 2023 and data was collected until 18 June 2023. Participants in this study had to be at least 18 years old, reside in South Africa, and consent to participate.

The R statistical software version 4.3.1 [29] was used to perform the statistical analysis for both descriptive (numbers and or percentages) and inferential statistics. Additionally, when appropriate, the Fischer’s test or Pearson’s chi-square test were used to determine whether two categorical variables were associated. The Wilcoxon rank sum test was employed to compare the median ages of the participant groups. All statistical tests were two-tailed, and the type-I error rate was set at 5% (α = 0.05).

## Results

A total of 374 survey responses were retrieved and 350 (completion rate of 93.6%) were analysed after excluding participants who did not reside in South Africa, records with missing ages and those who only completed the demographic section.

### Participants’ characteristics

The majority of participants (71.4%, *n* = 250; *N* = 350) were female with a median (IQR) age of 52 (38–62). The participants reported having a high Level of education, with only 0.3% (*n* = 1; *N* = 350) reporting no formal education and 86.0% (*n* = 301; *N* = 350) reporting tertiary education.

The majority of participants (91.4% *n* = 320; *N* = 350) lived in urban areas and all of South Africa’s provinces were represented in the study; the majority of participants resided in Gauteng (42.6%, *n* = 149; *N* = 350), the Western Cape (22.0%, *n* = 77; *N* = 350), the North West (9.4%, *n* = 33; *N* = 350), and KwaZulu-Natal (9.1%, *n* = 32; *N* = 350). The distribution of respondents in our study does not exactly match the distribution of the SA population [30]. However, a higher proportion of the respondents reside in some of the most populous provinces; notably KZN, WC, Gauteng. The characteristics of the participants are fully described in Table 1.

**Table 1:** Participant characteristics

Characteristic	Overall, *N* = 350^*1*^	Non-HCP, *N* = 248^*1*^	HCP *N* = 102^*1*^	*p*-value^2^
Sex				0.004
Female	250 (71.4%)	166 (66.9%)	84 (82.4%)	
Male	100 (28.6%)	82 (33.1%)	18 (17.6%)	
Age (year) (median, IQR)	52 (38–62)	53 (41–64)	46 (34–59)	0.002
Age Range (year)				0.014
19–30	40 (11.4%)	26 (10.5%)	14 (13.7%)	
31–40	61 (17.4%)	34 (13.7%)	27 (26.5%)	
41–50	66 (18.9%)	45 (18.1%)	21 (20.6%)	
51–60	86 (24.6%)	67 (27.0%)	19 (18.6%)	
61 and above	98 (27.7%)	77 (30.6%)	21 (20.6%)	
Area of residence				0.072
Rural	30 (8.6%)	17 (6.9%)	13 (12.7%)	
Urban	320 (91.4%)	231 (93.1%)	89 (87.3%)	
Province				-
Eastern Cape	13 (3.7%)	7 (2.8%)	6 (5.9%)	
Free State	17 (4.9%)	13 (5.2%)	4 (3.9%)	
Gauteng	149 (42.6%)	110 (44.4%)	39 (38.2%)	
KwaZulu-Natal	32 (9.1%)	21 (8.5%)	11 (10.8%)	
Limpopo	8 (2.3%)	3 (1.2%)	5 (4.9%)	
Mpumalanga	17(4.9%)	12 (4.8%)	5 (4.9%)	
North West	33 (9.4%)	19 (7.7%)	14 (13.7%)	
Northern Cape	4 (1.1%)	4 (1.6%)	0 (0.0%)	
Western Cape	77 (22.0%)	59 (23.8%)	18 (17.6%)	
Level of education				< 0.001
No formal education	1 (0.3%)	1 (0.4%)	0 (0.0%)	
Primary school	1 (0.3%)	1 (0.4%)	0 (0.0%)	
Secondary school	47(13.4%)	44 (17.7%)	3 (2.9%)	
Tertiary education	301 (86.0%)	202 (81.5%)	99 (97.1%)	

^1^Median (IQR); n (%)

^2^Wilcoxon rank sum test; Pearson’s Chi-squared test; Fisher’s exact test

HCP denotes participants who reported that they were healthcare professionals or who were enrolled in programs to become HCPs. Non- HCP refers to participants who are not healthcare professionals

Of the 350 participants, 29.1% (*n* = 102; *N* = 350) declared they were HCPs or pursuing a career in healthcare, and the majority of these participants (42.2%, *n* = 43; *N* = 102) were pharmacists. Nurses (27.5%, *n* = 28; *N* = 102), allied health professionals (7.8%, *n* = 8; *N* = 102), medical doctors (5.6%, *n* = 6; *N* = 102) and those who were classified as “other”—including epidemiologists, medical scientists, and paramedics (15.7%, *n* = 16; *N* = 102) were among the other health care professionals who took part in the study. To provide insights that consider participants’ presumptive medical training, the subsequent responses from the HCPs and non-HCPs groups will be provided separately where appropriate.

### Experience with ADEs and ADE reporting

Among the 288 participants who provided information on prior history with ADEs, 70.5% (*n* = 203; *N* = 288) declared to have experienced an ADE in the past. A higher proportion of non-HCP (74.3%, *n* = 150; *N* = 202) reported a history of ADEs compared with HCP (61.6%, *n* = 53; *N* = 86). The majority of the reported ADEs occurred after the use of medication (89.1%, *n* = 181; *N* = 203) with others occurring after the use of medical devices or during vaccination. Of the 59.1% (*n* = 120; *N* = 203) of participants who experienced ADEs and reported there was no difference (*p* > 0.9) between non-HCP (59.3%, *n* = 89; *N* = 150) and HCP (58.5%, *n* = 31; *N* = 53). Three of the 89 non-HCPs who reported ADEs but did not elaborate on their reasons for doing so, were excluded from analyses leaving only 117 participants. Of the 117 participants who reported the ADES the majority of these participants 80.3% (*n* = 94; *N* = 117) reported these events to doctors. Among participants who had a history of ADEs 39.3% (*n* = 59; *N* = 150) of non-HCPs and 37.7% (*n* = 20; *N* = 53) of the HCPs chose not to report the ADEs.

### Enabling factors of reporting ADEs

The participants who reported ADEs were asked for more information regarding the motivating factors behind their decision and 117 responses were obtained. The three main factors across all the participants that influenced (agree and strongly agree) their decision to report the ADE include: reporting could help to keep others safe (92.3%, *n* = 108; *N* = 117); being concerned about the adverse event they were experiencing (88.0%, *n* = 103; *N* = 117); and wanting to share their experience (82.9%, *n* = 97; *N* = 117). Only 4.3% (*n* = 5; *N* = 117) of participants were motivated by the need to obtain a refund of the money they had spent on the medicine. All the factors and the participants’ responses are listed in Table 2.

**Table 2:** Factors that influenced ADE reporting

Characteristic	Non-HCP *N* = 86^1^	HCP *N* = 31^*1*^
Was told by a healthcare professional to report		
Strongly Disagree	19 (22.1%)	5 (16.1%)
Disagree	30 (34.9%)	8 (25.8%)
Uncertain	17 (19.8%)	2 (6.5%)
Agree	14 (16.3%)	11 (35.5%)
Strongly Agree	6 (7.0%)	5 (16.1%)
Wanted a refund		
Strongly Disagree	38 (44.2%)	21 (67.7%)
Disagree	35 (40.7%)	7 (22.6%)
Uncertain	9 (10.5%)	2 (6.5%)
Agree	1 (1.2%)	0 (0.0%)
Strongly Agree	3 (3.5%)	1 (3.2%)
Worried about what was happening		
Strongly Disagree	1 (1.2%)	0 (0.0%)
Disagree	4 (4.7%)	3 (9.7%)
Uncertain	4 (4.7%)	2 (6.5%)
Agree	32 (37.2%)	8 (25.8%)
Strongly Agree	45 (52.3%)	18 (58.1%)
Wanted additional information		
Strongly Disagree	4 (4.7%)	4 (12.9%)
Disagree	7 (8.1%)	4 (12.9%)
Uncertain	8 (9.3%)	2 (6.5%)
Agree	36 (41.9%)	9 (29.0%)
Strongly Agree	31 (36.0%)	12 (38.7%)
Reporting is easy		
Strongly Disagree	15 (17.4%)	3 (9.7%)
Disagree	22 (25.6%)	5 (16.1%)
Uncertain	21 (24.4%)	5 (16.1%)
Agree	17 (19.8%)	11 (35.5%)
Strongly Agree	11 (12.8%)	7 (22.6%)
Wanted to share my experience		
Strongly Disagree	0 (0.0%)	0 (0.0%)
Disagree	8 (9.3%)	4 (12.9%)
Uncertain	8 (9.3%)	0 (0.0%)
Agree	39 (45.3%)	14 (45.2%)
Strongly Agree	31 (36.0%)	13 (41.9%)
Was upset about the situation		
Strongly Disagree	4 (4.7%)	3 (9.7%)
Disagree	14 (16.3%)	7 (22.6%)
Uncertain	6 (7.0%)	4 (12.9%)
Agree	33 (38.4%)	5 (16.1%)
Strongly Agree	29 (33.7%)	12 (38.7%)
Reporting can help to keep others safe		
Strongly Disagree	0 (0.0%)	1 (3.2%)
Disagree	2 (2.3%)	1 (3.2%)
Uncertain	4 (4.7%)	1 (3.2%)
Agree	26 (30.2%)	5 (16.1%)
Strongly Agree	54 (62.8%)	23 (74.2%)
It is my responsibility to report		
Strongly Disagree	0 (0.0%)	0 (0.0%)
Disagree	1 (1.2%)	1 (3.2%)
Uncertain	17 (19.8%)	1 (3.2%)
Agree	32 (37.2%)	10 (32%)
Strongly Agree	36 (41.9%)	19 (61.3%)

^1^n (%)

HCP denotes participants who reported that they were healthcare professionals or who were enrolled in programs to become HCPs. Non- HCP refers to participants who are not healthcare professionals

### Barriers to reporting ADEs

Participants who opted not to report the ADEs were questioned further about the barriers preventing them from doing so. One of the 59 non-HCPs who did not report the ADEs and did not give any reasons for doing so, was not included in the analysis. Three main barriers (agree and strongly agree) to reporting were cited by non-HCPs. Firstly, assuming that once medication was stopped, the side effects would go away (82.8.4%, *n* = 48; *N* = 58). Secondly, not knowing where to report (77.6%, *n* = 45; *N* = 58). Lastly, reading that the event they experienced was a side effect (72.4%, *n* = 42; *N* = 58). Similarly, three main barriers were cited by the HCPs. Firstly, assuming that once medication was stopped, the side effects would go away, (75.0%, *n* = 15; *N* = 20). Secondly, reading in the medicine leaflet that the event they had was a side effect (75.0%, *n* = 15; *N* = 20). Lastly, the belief that all medications have side effects implies it is not necessary to report (65.0%, *n* = 13; *N* = 20). The complete list of barriers and participants’ responses are listed in Table 3.

**Table 3:** Barriers to ADE reporting

Characteristic	Non-HCP *N* = 58^*1*^	HCP *N* = 20^*1*^
Did not know it had to be reported		
Strongly Disagree	4 (6.9%)	3 (15.0%)
Disagree	8 (13.8%)	9 (45.0%)
Uncertain	8 (13.8%)	3 (15.0%)
Agree	22 (37.9%)	3 (15.0%)
Strongly Agree	16 (27.6%)	2 (10.0%)
Did not know where to report it		
Strongly Disagree	5 (8.6%)	3 (15.0%)
Disagree	3 (5.2%)	6 (30.0%)
Uncertain	5 (8.6%)	1 (5.0%)
Agree	23 (39.7%)	6 (30.0%)
Strongly Agree	22 (37.9%)	4 (20.0%)
Reporting is too difficult		
Strongly Disagree	2 (3.4%)	0 (0.0%)
Disagree	4 (6.9%)	4 (20.0%)
Uncertain	23 (39.7%)	4 (20.0%)
Agree	12 (20.7%)	9 (45.0%)
Strongly Agree	17 (29.3%)	3 (15.0%)
Belief that all medications have side effects, so it is not necessary to report		
Strongly Disagree	8 (13.8%)	2 (10.0%)
Disagree	10 (17.2%)	4 (20.0%)
Uncertain	7 (12.1%)	1 (5.0%)
Agree	21 (36.2%)	10 (50.0%)
Strongly Agree	12 (20.7%)	3 (15.0%)
Assumed that once the medication was stopped, the side effects would go away		
Strongly Disagree	1 (1.7%)	3 (15.0%)
Disagree	4 (6.9%)	2 (10.0%)
Uncertain	5 (8.6%)	0 (0.0%)
Agree	35 (60.3%)	9 (45.0%)
Strongly Agree	13 (22.4%)	6 (30.0%)
No need to report because I stopped taking the medicine and the effect stopped		
Strongly Disagree	6 (10.3%)	4 (20.0%)
Disagree	12 (20.7%)	7 (35.0%)
Uncertain	8 (13.8%)	0 (0.0%)
Agree	22 (37.9%)	6 (30.0%)
Strongly Agree	10 (17.2%)	3 (15.0%)
Read that the event I had was a side effect		
Strongly Disagree	2 (3.4%)	2 (10.0%)
Disagree	6 (10.3%)	2 (10.0%)
Uncertain	8 (13.8%)	1 (5.0%)
Agree	28 (48.3%)	10 (50.0%)
Strongly Agree	14 (24.1%)	5 (25.0%)
Did not realise the event was related to medicine		
Strongly Disagree	15 (25.9%)	6 (30.0%)
Disagree	17 (29.3%)	12 (60.0%)
Uncertain	9 (15.5%)	0 (0.0%)
Agree	9 (15.5%)	2 (10.0%)
Strongly Agree	8 (13.8%)	0 (0.0%)

^1^n (%)

HCP denotes participants who reported that they were healthcare professionals or who were enrolled in programs to become HCPs. Non- HCP refers to participants who are not healthcare professionals

When participants who chose not to report their ADEs were asked if they were likely to report any future ADEs they might experience, 72.4% (*n* = 42; *N* = 58) of non-HCPs and 70.0% (*n* = 14; *N* = 20) of HCPs indicated they would report in the future while 24.1% (*n* = 14; *N* = 58) of non-HCPs and 25.0% (*n* = 5; *N* = 20) of HCPs continued to stand by their decision not to report.

## Discussion

This study described medicine users’ reporting habits and experiences, as well as the barriers and enablers to reporting ADEs in South Africa.

The majority of non-HCPs (74.3%, *n* = 150; *N* = 202) reported to have experienced ADEs in the past. While this result is similar to that of another South African study in which 72% of participants had experienced ADEs [31]. it is considerably higher than findings of other studies in which only half of participants reported that they experienced ADEs [6, 22, 24, 32, 33].

Occurrence of ADEs was significantly lower (*p* = 0.032) among HCPs (61.6%, *n* = 53; *N* = 86). This difference may be partly due to the fact that HCPs may have anticipated the events and thus not considered them to be adverse events [34]. The belief expressed by the HCPs (65.0%, *n* = 13; *N* = 20) that all medications have side effects so it is not necessary to report might act as a barrier to the recognition of ADEs by this group.

Compared to the reporting rate of a similar study performed in South Africa in which only 47% of the participants reported ADEs [31], in this study a greater proportion of participants who experienced ADEs reported them with 59.3% (*n* = 89; *N* = 150) of non-HCPs and 58.5% (*n* = 31; *N* = 53) of the HCPs indicated that they reported. In terms of the frequency of reporting ADEs, there was no appreciable distinction between the non-HCP and HCP groups (*p* > 0.9). This high rate of reporting was also seen in similar studies [6, 18, 22, 24, 32]. As seen in other studies [6, 18, 22, 24, 32, 35] participants of this study also preferred to report their ADEs to their doctors (80.3%, *n* = 94; *N* = 117) and this demonstrates that doctors are in a good position to influence reporting of ADEs.

The three main reasons given by study participants as to why they decided to report were: reporting can help keep others safe, being concerned about what was happening, and wanting to share their experience. All the factors and the participants’ responses are listed in Table 2. Similar studies where participants reported ADEs mostly with the intention of protecting others, highlighted the communal motives expressed by participants [11, 18, 20, 23, 35–37].

Overall, 39.3% (*n* = 59; *N* = 150) of non-HCPs and 37.7% (*n* = 20; *N* = 53) of the HCPs chose to not report ADEs that they experienced When asked for reasons why the ADEs were not reported, the most cited for the non-HCP group was assuming that once the medication was stopped, the side effects would go away; reading that the event they had was a side effect and not knowing where to report. The two barriers that the HCPs and non-HCPs shared were assuming that once the medication was stopped, the side effects would go away and reading that the event they had was a side effect. However, the third most cited barrier for HCPs was the belief that all medications have side effects, so it is not necessary to report. The complete list of barriers and participants’ responses are listed in Table 3.The barriers as identified in this study are consistent with previous research, which found that barriers to reporting ADEs included a lack of process awareness, knowledge that ADEs were listed as side effects, and expectations that the condition would stop when medication was stopped [11, 20, 23, 25, 26, 32, 33, 35, 38–42]. The barriers mentioned in this study suggest that participants were may not be fully aware of the reporting criteria.

Lack of awareness regarding what constitutes an ADE has been cited as one of the barriers to reporting ADEs [25, 26, 33]. Interestingly, study participants mentioned that knowing about and expecting ADEs acted as a barrier to reporting. Patients need to know that even though ADEs may be expected or listed in the package insert, they should still be reported. Awareness campaigns aimed at improving ADE reporting must also emphasize what information should be provided.

Concerning the willingness of participants who had not previously reported ADEs to do so in the future, it is encouraging that 72.4% (*n* = 42; *N* = 58) of non-HCPs and 70.0% (*n* = 14; *N* = 20) of HCPs indicated they would report ADEs. Nonetheless, 24.1% (*n* = 14; *N* = 58) of non-HCPs and 25.0% (*n* = 5; *N* = 20) of HCPs continued to support their decision not to report.

One of the limitations of the recruitment and data-collection method is that only participants who had access to the Netwerk24/News24 platform during the period of advertisement accessed the invitation to participate in the study. Another limitation relates to the fact that the data collection instrument was only available in English. A further limitation of the study is that the study invited respondents to take part in a survey on medication safety, implying a likelihood of attracting participants who had experienced adverse effects or had heightened concerns about medication safety. Another limitation was that even though participants came from all provinces in South Africa, the generalisability of the results is limited by the small sample size and the predominance of participants who resided in urban areas as well as the high proportion (85.8%) of study participants who had completed tertiary education. It is worth noting that South Africa’s post-secondary school completion rate is currently 18.8% [43]. Lastly recall bias could affect the study results as the survey relied on the participant’s ability to remember past experiences.

Nevertheless, despite the aforementioned limitation, the study was able to provide the information required to fulfil the study’s objectives and offer insights into the current ADE reporting situation in South Africa. The HCP’s perspectives as medicine users offered information that can be applied further to initiatives aimed at enhancing patient ADE reporting in South Africa.

## Conclusions

High reporting rates for ADEs were observed among participants and doctors were the preferred recipients of these reports. In terms of reporting ADEs and the justifications for doing so, we discovered that HCPs and non-HCPs were comparable. We identified several barriers to reporting, and one of them is awareness of the anticipated ADEs. This study presents information on the barriers and facilitators to reporting ADEs, which can be used to enhance awareness-raising campaigns aimed at improving ADE reporting in South Africa.

## Data Availability

The datasets used and/or analysed during the current study are available from the corresponding author upon reasonable request.

## Abbreviations

ADE: Adverse Drug Event
HCP: Healthcare professional
IQR: Interquartile Range
SAHPRA: South African Health Product Regulatory Authority
WHO: World Health Organization
